# Pay No License Fees

**Published:** 1903-09-15

**Authors:** J. N. Crouse

**Affiliations:** Chicago


### PAY NO LICENSE FEES.

During the past few days members of the Protective Association throughout the East have been forwarding to us a letter sent them by Mrs. Mary Sheffield, in which it is claimed that Mrs. Sheffield is practically the sole owner of the International Tooth Crown Co., that she has full power to make all settlements for claims for damages, and to grant licenses under the numerous patents owned by that company, and that she will do so for the sum of $25.00.

We advise you to pay no money whatever to the International Tooth Crown Co., because of this recent letter or for any other reason. The Protective Association has fought that company for about sixteen years in nearly all the courts of this country, and that company has not in any instance obtained a dollar from any member of the Association in any such litigation, and in our opinion it never will. There is no possible danger of the Crown Company ever beginning another suit against any member of our Association, but if a suit should be brought there is not the slightest chance that the company would be successful. All suits against members of the Association in good standing will be defended by the Association without cost to them as heretofore. By "good standing" we mean those who have paid $20.00, covering the membership fee and assessment.

We do not care to go into any details of the Association's plans at this time, but we earnestly advise you not to contribute at the last moment this sum of $25.00 to the Crown Company, when we have been victorious for so many years and have kept that company from harassing or collecting money from you. We would also call your attention to the fact that the sum now demanded by the Crown Company is $5.00 more than the entire amount of money you have paid the Association for defending and protecting you in the courts for the last sixteen years against this and several other companies.

Yours very truly,

THE DENTAL PROTECTIVE ASSO.

Chicago, June 19, 1903. J. N. Crouse, *Chairman.*,

